# High-Mobility Inkjet-Printed Indium-Gallium-Zinc-Oxide Thin-Film Transistors Using Sr-Doped Al_2_O_3_ Gate Dielectric

**DOI:** 10.3390/ma12060852

**Published:** 2019-03-13

**Authors:** Seungbeom Choi, Kyung-Tae Kim, Sung Kyu Park, Yong-Hoon Kim

**Affiliations:** 1 SKKU Advanced Institute of Nanotechnology (SAINT), Sungkyunkwan University, Suwon 16419, Korea; oyenice@skku.edu; 2 School of Electrical and Electronic Engineering, Chung-Ang University, Seoul 06974, Korea; 4444812@naver.com; 3 School of Advanced Materials Science and Engineering, Sungkyunkwan University, Suwon 16419, Korea

**Keywords:** metal-oxide semiconductors, thin-film transistors, inkjet printing, high-k dielectric, high mobility

## Abstract

In this paper, we demonstrate high-mobility inkjet-printed indium-gallium-zinc-oxide (IGZO) thin-film transistors (TFTs) using a solution-processed Sr-doped Al_2_O_3_ (SAO) gate dielectric. Particularly, to enhance to the electrical properties of inkjet-printed IGZO TFTs, a linear-type printing pattern was adopted for printing the IGZO channel layer. Compared to dot array printing patterns (4 × 4 and 5 × 5 dot arrays), the linear-type pattern resulted in the formation of a relatively thin and uniform IGZO channel layer. Also, to improve the subthreshold characteristics and low-voltage operation of the device, a high-*k* and thin (~10 nm) SAO film was used as the gate dielectric layer. Compared to the devices with SiO_2_ gate dielectric, the inkjet-printed IGZO TFTs with SAO gate dielectric exhibited substantially high field-effect mobility (30.7 cm^2^/Vs). Moreover, the subthreshold slope and total trap density of states were also significantly reduced to 0.14 V/decade and 8.4 × 10^11^/cm^2^·eV, respectively.

## 1. Introduction

Amorphous oxide semiconductor (AOS)-based thin-film transistors (TFTs) have received a considerable interest in the fields of displays, sensors, and wearable electronics owing to their high carrier mobility, good operational stability and flexibility, and large-area scalability [1,2,3,4,5]. Particularly, solution-processed AOS TFTs have been intensively studied lately [6,7,8,9], by the fact that their electrical performances are now competing with those fabricated by vacuum-deposition methods such as sputtering. In the fabrication of AOS-based TFTs, the AOS channel layer should be patterned in order to isolate each TFT device and reduce the leakage between the devices. Typically, to pattern the AOS channel layer, conventional photolithography and etching processes are used. A self-patterning method which utilizes the surface energy difference can also be used to pattern the AOS channel layer without using the photolithography process [10,11,12], however, this may require wet chemical processes to obtain the surface energy contrast. Direct printing methods such as inkjet printing, on the other hand, allow a simple and efficient way to pattern the AOS channel layer, by directly forming the channel layer on the substrate.

Previously, several different approaches for fabricating AOS TFTs by inkjet printing have been demonstrated [13,14,15,16,17]. Zhang et al. proposed a self-aligned inkjet printing process to fabricated small-channel indium-gallium-zinc-oxide (IGZO) TFTs using a polymer bank structure [14]. Li et al. demonstrated self-aligned short-channel InScO TFTs using coffee stripe dewetting method [15], where a hydrophobic polymer such as CYTOP was inkjet-printed to prepare a coffee stripe and used to pattern the gate electrode and form the self-aligned structure. In addition, Li et al. also demonstrated the fabrication of inkjet-printed InGaO TFTs by using a surface-energy patterned structure [16]. Here, the inkjet printing of pure solvent on pre-coated hydrophobic polymer allowed the modification of surface energy selectively, which induced controlled formation of inkjet-printed channel layer [16]. Although these previous reports on inkjet-printed AOS TFTs provide new approaches to fabricate TFTs via using direct printing method, the electrical properties such as field-effect mobility and subthreshold characteristics still need improvements. In addition, in inkjet-printed AOS TFTs, the coffee-ring effect [18] can cause problems, such as irregular channel thickness and morphology, resulting in poor and non-uniform electrical properties. Therefore, a new approach to reduce the influence of coffee-ring effect needs to be developed for high-performance inkjet-printed AOS TFTs. Additionally, for high-performance and low-voltage operation of oxide TFTs, high-*k* gate dielectrics such as Zr, La, or Sr-doped Al_2_O_3_ (SAO) can be promising [19,20,21]. Particularly, the Sr doping in Al_2_O_3_ may also improve the leakage current and subthreshold characteristics [21].

Here, we demonstrate high-mobility inkjet-printed IGZO TFTs using a solution-processed SAO gate dielectric. Specifically, in order to obtain high saturation field-effect mobility and to reduce the operating voltage, a high-*k* and thin (~10 nm) SAO gate dielectric was used. This allowed substantially improved subthreshold characteristics of the device. In addition, we adopted a linear-type printing pattern to inkjet print the IGZO channel layer. Compared to the 4 × 4 or 5 × 5 dot array printing patterns, the linear-type printing pattern facilitated the formation of thin and uniform channel layer in the active channel region, allowing high field-effect mobility (>30 cm^2^/Vs) and low subthreshold slope (*SS*) (0.14 V/decade).

## 2. Experimental Procedure

For inkjet printing of the channel layer, an IGZO precursor solution was prepared by dissolving indium nitrate hydrate (In(NO_3_)_3_·*x*H_2_O), gallium nitrate hydrate (Ga(NO_3_)_3_·*x*H_2_O), and zinc nitrate hydrate (Zn(NO_3_)_2_·*x*H_2_O) in 2-methoxyethanol with a molar ratio of 6.8:1.0:2.2 (In:Ga:Zn). The total molar concentration of the precursor solution was 0.25 M. All the metallic precursors and the solvent were purchased from Sigma-Aldrich (St. Louis, MO, USA). After dissolving, the solution was stirred for ~12 h at 75 °C before use. To fabricate IGZO TFTs by inkjet printing, a heavily doped p-type Si wafer having a 200 nm-thick SiO_2_ layer was used as a substrate. The p-type Si wafer also served as the gate electrode. The Si/SiO_2_ wafer was sequentially cleaned in acetone, isopropanol alcohol, and UV/ozone (UVC-30, Jaesung Engineering Co., Anyang, Korea) before printing the IGZO film. For the formation of the IGZO channel layer, an inkjet printing system (DMP-2850, Fujifilm, Santa Clara, CA, USA) equipped with a cartridge DMC-11610 (10 pL, Dimatix, Santa Clara, CA, USA) was used. The jetting waveform used for printing was 0 V (7.618 μs)→15 V (14.08 μs, slew rate 1.99 V/μs)→0 V (5.824 μs, slew rate 0.5 V/μs). During the printing, the head and plate temperatures were set at 35 °C and 50 °C, respectively. Several different printing patterns were used to print the IGZO channel layer, including 4 × 4 (drop spacing = 10 μm) and 5 × 5 (drop spacing = 5 μm) dot array printing patterns and a linear-type printing pattern (drop spacing = 10 μm, total of 121 drops). After printing the IGZO precursor solution, the substrate was thermally annealed at 350 °C for 1 h in ambient air. For source/drain (S/D) electrodes, a 75 nm-thick aluminum (Al) layer was deposited by thermal evaporation method. For the fabrication of SAO gate dielectric, an SAO precursor solution was prepared by dissolving aluminum nitrate nonahydrate (Al(NO_3_)_3_·9H_2_O) and strontium nitrate (Sr(NO_3_)_2_) in 2-methoxyethanol with a molar ratio of 9.5:0.5 (Al:Sr). The total molar concentration of the precursor solution was 0.15 M. Then, the SAO precursor solution was spin-coated on a Si wafer and thermally annealed at 350 °C for 1 h. The thermally annealed SAO film had a thickness and dielectric constant of ~10 nm and ~7, respectively. The patterning of Al S/D electrodes was carried out by using a metal shadow mask. The transfer and output characteristics of IGZO TFTs were measured by using a semiconductor parameter analyzer (4155C, Agilent, Santa Clara, CA, USA). The film morphology and the thickness profiles of inkjet-printed IGZO TFTs were analyzed by using a confocal microscope (VK-9710, Keyence, Osaka, Japan) and a stylus profiler (DektakXT, Bruker, Billerica, MA, USA).

## 3. Results and Discussion

**Figure 1:**
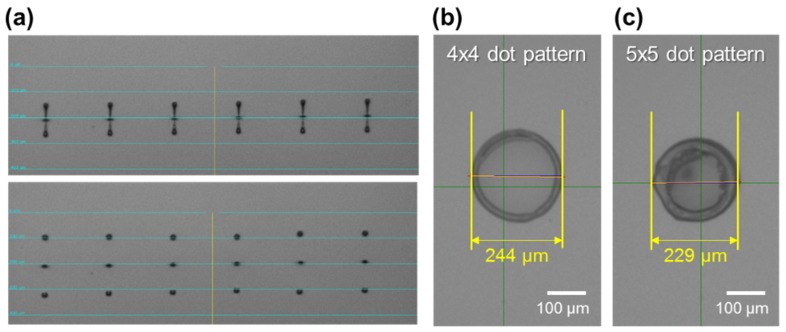
(**a**) Optical images of inkjet-printed indium-gallium-zinc-oxide (IGZO) droplets from inkjet head, and optical images of IGZO channel layers printed by (**b**) 4 × 4, and (**c**) 5 × 5 dot array patterns. Here, the drop spacing between the dots were 10 μm and 5 μm for 4 × 4 and 5 × 5 dot array, respectively.

**Figure 2:**
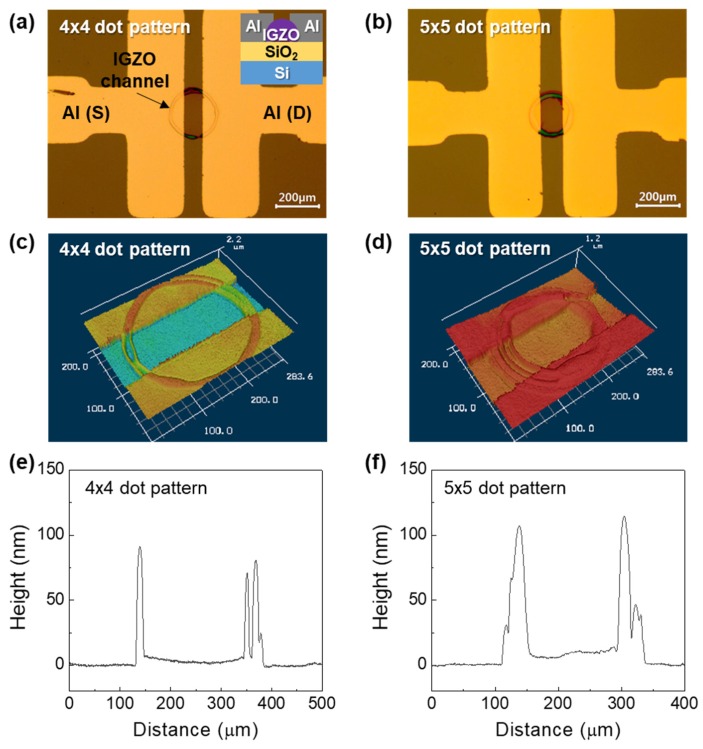
Optical microscope images of inkjet-printed IGZO thin-film transistors (TFTs) printed by (**a**) 4 × 4, and (**b**) 5 × 5 dot array patterns. The inset in (**a**) shows the schematic device structure of an IGZO TFT with SiO_2_ gate dielectric. Confocal microscope images of inkjet-printed IGZO channel layer printed by (**c**) 4 × 4, and (**d**) 5 × 5 dot array patterns. Thickness profiles of inkjet-printed IGZO channel layer printed by (**e**) 4 × 4, and (**f**) 5 × 5 dot array patterns.

To form the IGZO channel layer by inkjet printing, 4 × 4 and 5 × 5 dot array printing patterns were first employed. For a 4 × 4 dot array, the drop spacing between each drop was 10 μm in vertical and lateral directions. Therefore, a total of 16 drops was used to form each channel pattern. For 5 × 5 dot array, the drop spacing was 5 μm in vertical and lateral directions, giving a total of 25 drops per each channel pattern. A single drop can be more favorable for practical application, however, in our case, when a low molar concentration (0.25 M) IGZO precursor solution is used, the thickness of the channel layer especially at the center region was very thin and the device showed relatively poor electrical characteristics. Figure 1a shows the optical image of IGZO droplets jetted from the inkjet head. It can be seen that the droplets shapes and sizes are relatively uniform, and stable jetting was possible. Figure 1b,c show the optical images of inkjet-printed IGZO channel layers printed by 4 × 4 and 5 × 5 dot array pattern, respectively. The diameters of the printed channel layers were 244 μm and 229 μm for 4 × 4 and 5 × 5 dot array patterns, respectively. Using the inkjet-printed IGZO film as a channel layer, top-contact IGZO TFTs were fabricated by depositing Al S/D electrodes on top of the IGZO channel layer. Figure 2a,b show the inkjet-printed IGZO TFTs with 4 × 4 and 5 × 5 dot array patterns. It is observed that the IGZO channels are well positioned between the S/D electrodes. Figure 2c,d and Figure 2e,f show the confocal microscope images and thickness profiles of inkjet-printed IGZO channel layer printed by 4 × 4 and 5 × 5 dot array patterns. Evidence of the coffee-ring effect were observed in which the films are thicker at the outer region. At the center regions, the film thickness was 2–7 nm (4 × 4 dot array) and 5–12 nm (5 × 5 dot array), while the outer regions had thickness of ~100 nm.

**Figure 3:**
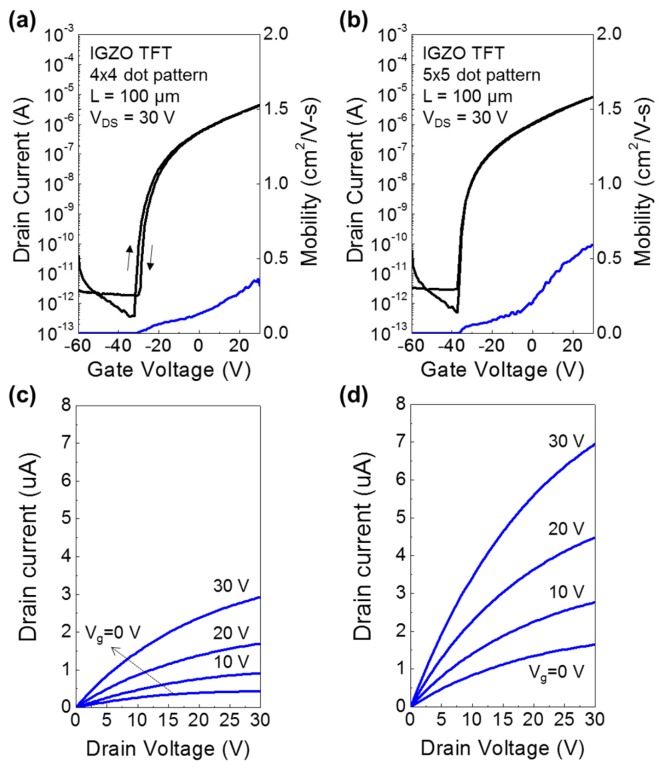
Transfer characteristics of IGZO TFTs inkjet-printed with (**a**) 4 × 4, and (**b**) 5 × 5 dot array patterns. Output curves of IGZO TFTs inkjet-printed with (**c**) 4 × 4, and (**d**) 5 × 5 dot array printing patterns.

Using the inkjet-printed IGZO channel layers, TFTs with top-contact structure were fabricated and their transfer characteristics were analyzed as shown in Figure 3a,b, with 4 × 4 and 5 × 5 dot array printing patterns, respectively. Also, Figure 3c,d show the corresponding output characteristics of IGZO TFTs for 4 × 4 and 5 × 5 dot array patterns, respectively. The saturation field-effect mobility was extracted by using the following Equation,

(1) $$I_{D}=\;\frac{W}{2L}\mu C_{i}{\left(V_{G}-V_{th}\right)}^{2}$$

where, *I_D_* is the drain current, *L* and *W* are the channel length and width, respectively, *C_i_* is the areal capacitance of the gate dielectric, and *V_g_* is gate voltage and *V_th_* is threshold voltage. In the case of the 4 × 4 dot array, the TFT device exhibited a saturation field-effect mobility of 0.23 ± 0.073 cm^2^/Vs, while the TFT with 5 × 5 dot array exhibited a saturation mobility of 0.37 ± 0.105 cm^2^/Vs. Compared to those fabricated using spin coating method, the mobilities are relatively low [9]. However, the on/off ratio value (~10^7^) was similar to those of spin-coated TFT devices. Moreover, the inkjet-printed TFT devices exhibited large negative threshold voltage (*V_th_*). Particularly, the *V_th_* values for TFTs shown in Figure 3a,b were −27.8 V and −33.8 V, respectively. The large negative *V_th_* can be attributed to the thick outer channel regions which lie inside the channel region (region between the S/D electrodes). In addition, the low dielectric constant (~3.9) and the large thickness of the SiO_2_ gate dielectric layer also contributes to the large negative *V_th_*. Furthermore, as shown in the output characteristics (Figure 3c,d), the devices showed large leakage currents even when the gate bias was 0 V.

**Figure 4:**
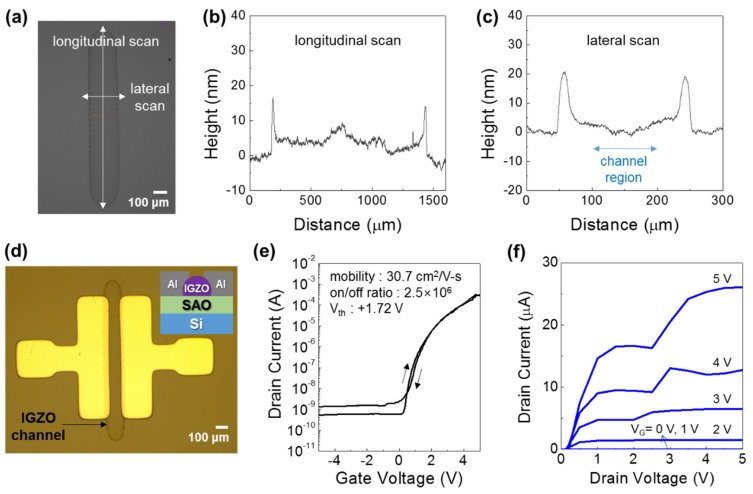
(**a**) An optical image of inkjet-printed IGZO channel layer with linear-type drop pattern. Thickness profiles of an inkjet-printed IGZO channel layer, (**b**) longitudinal scan, and (**c**) lateral scan. (**d**) An optical image, (**e**) transfer characteristics, and (**f**) output characteristics of an inkjet-printed IGZO TFT with linear-type pattern.

In this perspective, to enhance the electrical properties of inkjet-printed IGZO TFTs, the outer channel thickness should be reduced and a thin gate dielectric layer with high dielectric constant should be adopted. To meet these requirements, we modified the arrayed printing pattern into linear-type pattern, and a solution-processed SAO film was used as a gate dielectric layer instead of SiO_2_ film. At first, the inkjet printing pattern was changed to linear-type as shown in Figure 4a. Here, the drop spacing distance was 40 μm with a total of 31 drops. This resulted in the formation of an IGZO channel layer having a dimension of ~220 μm × 1200 μm with relatively uniform shape. More importantly, the center region of the printed channel layer was relatively uniform, having a thickness of 5–10 nm as shown in Figure 4b,c. Although the outer regions of the printed channel layer are relatively thicker, its influence on the electrical properties of TFTs may not be significant since these regions lie outside the channel region (region between the S/D electrodes), as indicated in Figure 4c (lateral scan thickness profile).

Using the inkjet-printed IGZO channel layer having linear pattern, IGZO TFTs were fabricated by employing a solution-processed SAO gate dielectric. Here, for SAO gate dielectric, a small amount (5 at.%) of strontium was added to the Al_2_O_3_ to increase the dielectric constant of the film [21]. The SAO gate dielectric layer thermally annealed at 350 °C had the thickness and dielectric constant of ~10 nm and ~7, respectively. Figure 4c,d shows an optical microscope image and the transfer characteristics of inkjet-printed IGZO TFT with SAO gate dielectric. The IGZO TFT exhibited a clear modulation of current in gate bias (*V_g_*) range of −5 V to +5 V. Compared to those fabricated on SiO_2_ gate dielectric and with arrayed printing patterns, the operation voltage and the *V_th_* value (+1.72 V) were much lower owing to the large dielectric constant and thin thickness of the SAO gate dielectric. Also, the inkjet-printed IGZO TFTs showed relatively high saturation field-effect mobility and on/off ratio of 30.7 cm^2^/Vs and 2.5 × 10^6^, respectively. In addition, the TFT device showed negligible hysteresis in the transfer curves, indicating that the IGZO–SAO interface has relatively low interfacial states. From the transfer characteristics, the *SS* and the total trap density of states (*N_t_*) were obtained using the following Equations [22],

(2) $$SS=\frac{dV_{g}}{d\left(logI_{d}\right)}$$

(3) $$SS=\frac{kT\ln10}{e}\left[1+\frac{e^{2}}{C_{i}}N_{t}\right]$$

where, *k* is the Boltzmann constant, and *e* is the elementary charge. The obtained *SS* and *N_t_* values were 0.14 V/decade and 8.4 × 10^11^/cm^2^·eV, respectively. As compared to the devices fabricated on SiO_2_ gate dielectric using dot array printed IGZO channel layers, the *SS* and *N_t_* are greatly improved possibly owing to the high *C_i_* value of the device. In fact, the inkjet-printed IGZO TFTs with 4 × 4 dot array pattern exhibited *SS* and *N_t_* values of 1.68 V/decade and 2.9 × 10^12^/cm^2^·eV, respectively, and the IGZO TFTs with 5 × 5 dot array pattern exhibited *SS* and *N_t_* values of 1.27 V/decade and 2.2 × 10^12^/cm^2^·eV, respectively. In overall, by using a line-type pattern of inkjet-printed IGZO channel layer and solution-processed high-*k* SAO gate dielectric, the electrical properties of the TFTs including saturation mobility, on/off ratio and *SS* were significantly improved.

## 4. Conclusions

Here, we demonstrated high-mobility inkjet-printed IGZO TFTs using a solution-processed SAO gate dielectric. To obtain high saturation field-effect mobility and to reduce the operating voltage, a high-*k* and thin SAO gate dielectric was used. In addition, we adopted a linear-type printing pattern to inkjet print the IGZO channel layer and compared to the dot array printing patterns. The linear-type printing pattern facilitated the formation of a thin and uniform channel layer in the active channel region, resulting in high mobility TFT devices via the inkjet printing method.
